# Impact of indoor temperature instability on diurnal and day-by-day variability of home blood pressure in winter: a nationwide Smart Wellness Housing survey in Japan

**DOI:** 10.1038/s41440-021-00699-x

**Published:** 2021-07-29

**Authors:** Wataru Umishio, Toshiharu Ikaga, Kazuomi Kario, Yoshihisa Fujino, Masaru Suzuki, Shintaro Ando, Tanji Hoshi, Takesumi Yoshimura, Hiroshi Yoshino, Shuzo Murakami, on behalf of the SWH survey group

**Affiliations:** 1 Department of Architecture and Building Engineering, School of Environment and Society, Tokyo Institute of Technology, Ookayama, Meguro-ku, Tokyo Japan; 2 Department of System Design Engineering, Faculty of Science and Technology, Keio University, Yokohama, Kanagawa Japan; 3 Department of Cardiology, Jichi Medical University School of Medicine, Shimotsuke, Tochigi Japan; 4 Department of Environmental Epidemiology, Institute of Industrial Ecological Sciences, University of Occupational and Environmental Health, Kitakyushu, Fukuoka Japan; 5 Department of Emergency Medicine, Ichikawa General Hospital, Tokyo Dental College, Ichikawa, Chiba Japan; 6 Department of Architecture, Faculty of Environmental Engineering, University of Kitakyushu, Kitakyushu, Fukuoka Japan; 7 Tokyo Metropolitan University, Hachioji, Tokyo Japan; 8 University of Occupational and Environmental Health, Kitakyushu, Fukuoka Japan; 9 Tohoku University, Sendai, Miyagi Japan; 10 Institute for Building Environment and Energy Conservation, Kojimachi, Chiyoda-ku, Tokyo Japan

**Keywords:** Day-by-day variability, Diurnal variability, Home blood pressure, Housing, Indoor temperature

## Abstract

Home blood pressure (HBP) variability is an important factor for cardiovascular events. While several studies have examined the effects of individual attributes and lifestyle factors on reducing HBP variability, the effects of living environment remain unknown. We hypothesized that a stable home thermal environment contributes to reducing HBP variability. We conducted an epidemiological survey on HBP and indoor temperature in 3785 participants (2162 households) planning to have their houses retrofitted with insulation. HBP was measured twice in the morning and evening for 2 weeks in winter. Indoor temperature was recorded with each HBP observation. We calculated the morning-evening (ME) difference as an index of diurnal variability and the standard deviation (SD), coefficient of variation (CV), average real variability (ARV) and variability independent of the mean (VIM) as indices of day-by-day variability. The association between BP variability and temperature instability was analyzed using multiple linear regression models. The mean ME difference in indoor/outdoor temperature (a decrease in temperature overnight) was 3.2/1.5 °C, and the mean SD of indoor/outdoor temperature was 1.6/2.5 °C. Linear regression analyses showed that the ME difference in indoor temperature was closely correlated with the ME difference in systolic BP (0.85 mmHg/°C, *p* < 0.001). The SD of indoor temperature was also associated with the SD of systolic BP (0.61 mmHg/°C, *p* < 0.001). The CV, ARV, and VIM showed similar trends as the SD of BP. In contrast, outdoor temperature instability was not associated with either diurnal or day-by-day HBP variability. Therefore, residents should keep the indoor temperature stable to reduce BP variability.

## Graphical abstract

**Figure:**
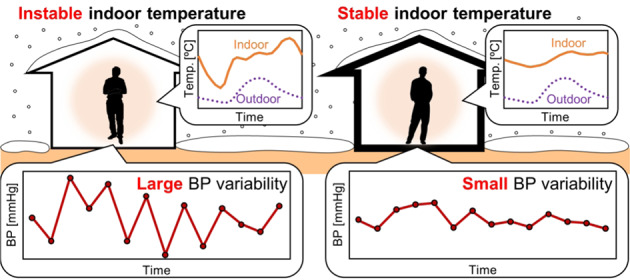
Diurnal HBP variability (Morning-Evening difference) and day-by-day HBP variabilities (SD, CV, ARV, and VIM) were significantly associated with indoor temperature instability but not with outdoor temperature instability.

## Introduction

Home blood pressure monitoring (HBPM) is currently one of the standard methods used in the diagnosis and management of hypertension. The American [1], European [2], and Japanese guidelines [3] for hypertension recommend out-of-office blood pressure (BP) monitoring to minimize the white coat effect, observer bias, and measurement errors. HBPM is also better tolerated and more widely used than ambulatory blood pressure monitoring [4]. Previous studies have revealed a strong association between home BP (HBP) level and cardiovascular disease (CVD) events [5, 6].

Aside from HBP level, HBP variability is also an important factor when evaluating the risk of CVD events. BP can exhibit different variabilities with different time phases (beat-by-beat, diurnal, day-by-day, seasonal, and yearly variability), and a CVD event may occur when all BP variabilities are synchronized to generate a large dynamic surge in BP [7]. HBPM is useful for assessing a wide range of BP variabilities, including diurnal variability, as indicated by morning-evening difference (ME difference), and day-by-day variability, as indicated by the standard deviation (SD), coefficient of variation (CV), average real variability (ARV), and variability independent of the mean (VIM) measurements. Several studies have indicated that there are additional benefits to reducing the ME difference [8] and day-by-day variability indices described above [9, 10], independent of HBP level. Reducing HBP variability is therefore valuable for preventing CVD events.

Several studies [11–13] have examined the effects of individual attributes and lifestyle factors for reducing HBP variability. In contrast, however, the effects of the living environment remain unknown. Given that previous research has shown an association between indoor temperature and HBP level [14, 15], we hypothesized that a stable indoor thermal environment at home would contribute to reducing HBP variability. One of the primary factors of indoor temperature instability is the thermal insulation level of houses. Houses with little thermal insulation are easily affected by outdoor weather conditions, which results in unstable indoor temperatures. In contrast, houses with high amounts of thermal insulation are able to maintain the indoor temperature within an appropriate range. Therefore, housing quality may play an essential role in reducing HBP variability. In Japan, an estimated 39% of existing houses are uninsulated [16], making this an important setting in which to examine the association between indoor temperature instability and HBP variability.

We conducted a nationwide prospective intervention trial named the “Smart Wellness Housing (SWH) survey,” which aimed to quantitatively evaluate the relationship between HBP and indoor temperature in Japan. In our previous paper, we focused on HBP level [14]. In this paper, we investigated the association between HBP variability and indoor temperature instability based on the results of the baseline (before intervention) survey.

## Methods

The authors declare that the data supporting the findings of this study are provided within this article and its online-only Data Supplement. The study was conducted according to the principles of the Declaration of Helsinki. The study protocol and informed consent procedure were approved by the ethics committee of the Hattori Clinic Ethics Review Board (Approval No. S1410-J03). This review board consists of experts in medicine, bioethics, and law and is certified by the Ministry of Health, Labour and Welfare (Accreditation No. CRB3180027). All of the participants provided written informed consent to participate and to have their data published. This study is registered at http://www.umin.ac.jp/ctr/ (Trial No. UMIN000030601).

### Study design

The study design of the SWH survey is reported elsewhere [14]. This survey was conducted as a nonrandomized controlled trial with groups defined according to participants’ choice on whether to have their houses retrofitted with insulation. Participants were recruited by construction companies throughout all 47 prefectures in Japan. Inclusion criteria were (1) intention to have their houses retrofitted with insulation, (2) age over 20 years, and (3) house prior to renovation did not meet the S (Supreme) standards of the “Act on the Promotion of Dissemination of Long-Lasting Quality Housing” in Japan [17]. The SWH survey was initiated in the winter of 2014 and obtained data for a total of six winter periods (2014–2019) up to January 2021. In this paper, we performed a cross-sectional analysis of the data obtained in the baseline (before insulation retrofitting) survey in winter.

### Home blood pressure and other measurements

Methods for measuring HBP and other parameters are also reported elsewhere [14]. Briefly, HBP was measured twice after awaking in the morning (after urination, before dosing, and before breakfast) and twice before getting into bed in the evening, in accordance with the current guidelines [18]. The two HBP observations in each morning/evening were averaged and used in the following calculation of HBP variability. HBP was measured in the living room for 2 weeks in the sitting position using an automatic oscillometric device (HEM-7251G; Omron Healthcare Co., Ltd., Kyoto). HBP data were automatically stored with indoor ambient temperature data. Room temperature at 1.0 m above the floor was also measured in the living room at 10-min intervals (TR-72wf; T&D Corp., Nagano). Outdoor temperature at 60-min intervals was obtained from the closest local meteorological observatory to each participant’s house. A questionnaire survey was also conducted, which covered individual attributes, such as age, sex, weight, and household income; lifestyle indicators, such as eating habits, smoking, and alcohol consumption; and diseases associated with hypertension. Furthermore, a diary survey was conducted in which participants provided their daily time spent at home, sleep quality, and duration.

To evaluate diurnal HBP variability, we calculated the ME difference (morning BP−evening BP; Supplementary Fig. 1). To evaluate day-by-day HBP variability, we calculated the SD, CV (100 × SD/mean of each participant’s BP), ARV (average absolute difference between successive BP measurements), and VIM (an index with no correlation with mean BP level). The morning-evening average (ME average) BP was used as the BP value of each day to calculate the above day-by-day variability indices. We removed participants with less than 5 days of data according to the current guidelines [18]. We also excluded HBP observations that were measured after a lapse of 14 days. The details of these calculations are shown in Supplementary Fig. 2. These variability indices have been used in previous HBP variability studies [9, 10]. We also determined the ME difference in the indoor and outdoor temperatures (evening temperature–morning temperature) to evaluate diurnal temperature instability. Indoor ambient temperature, which was stored with the HBP value at the same time as each morning/evening measurement, and outdoor temperature, the recorded time of which was the closest to each HBP measurement, were used in the calculation. The SD of the indoor and outdoor temperature to evaluate day-by-day temperature instability was calculated based on the ME average temperature of each day.

### Statistical analysis

Multiple linear regression analysis was used to examine the association between HBP variability and indoor temperature instability. To examine diurnal variability, Model 1 was developed to include the ME difference in BP as a dependent variable and the ME difference in indoor and outdoor temperature as independent variables. To examine day-by-day variability, four models were developed: Model 2-1 included the SD, Model 2-2 included the CV, Model 2–3 included the ARV, and Model 2–4 included the VIM of 2-week HBP measurements as dependent variables and the SD of indoor and outdoor temperature as independent variables. Both Models 1 and 2 were adjusted for the following variables: average indoor ambient temperature and outdoor temperature; age; sex; body mass index (BMI); high household income (≥6 or <6 million JPY); the salt check sheet score; [19] vegetable consumption (eating vegetables regularly or not); current smoker; current drinker of alcohol; regular exercise (≥30 min/opportunity and ≥2 opportunities/week or not); and use of antihypertensive drugs. The salt check sheet (0–35 points) is a simple questionnaire about salt intake that is completed by checking the frequency of eating salty food, which significantly correlates with 24-h urinary salt excretion levels. We compared the regionally averaged salt check sheet score in the present survey with the salt intake in the National Health and Nutrition Survey to check the validity of these scores beforehand (Supplementary Table 1). Given that previous studies indicated that morning blood pressure becomes high when sleep quality is low [20], we additionally adjusted sleep quality and duration. Averages of 2 sleep indices were input to both models. The SDs of two indices were input to Model 2 because day-by-day HBP variability was thought to be affected by day-by-day variability in sleep quality and duration. These variables were selected as factors related to hypertension based on the Guidelines for the Management of Hypertension 2014 (JSH2014) [21]. Furthermore, we adjusted the number of HBP measurement days in Model 2 because it might affect the day-by-day variability indices. All P values were two sided, and a two-sided P value less than 0.05 was considered statistically significant. All analyses were performed using SPSS Ver. 26 (SPSS Inc., Chicago, Illinois, USA).

## Results

### Study profile of the baseline survey in winter

**Fig. 1:**
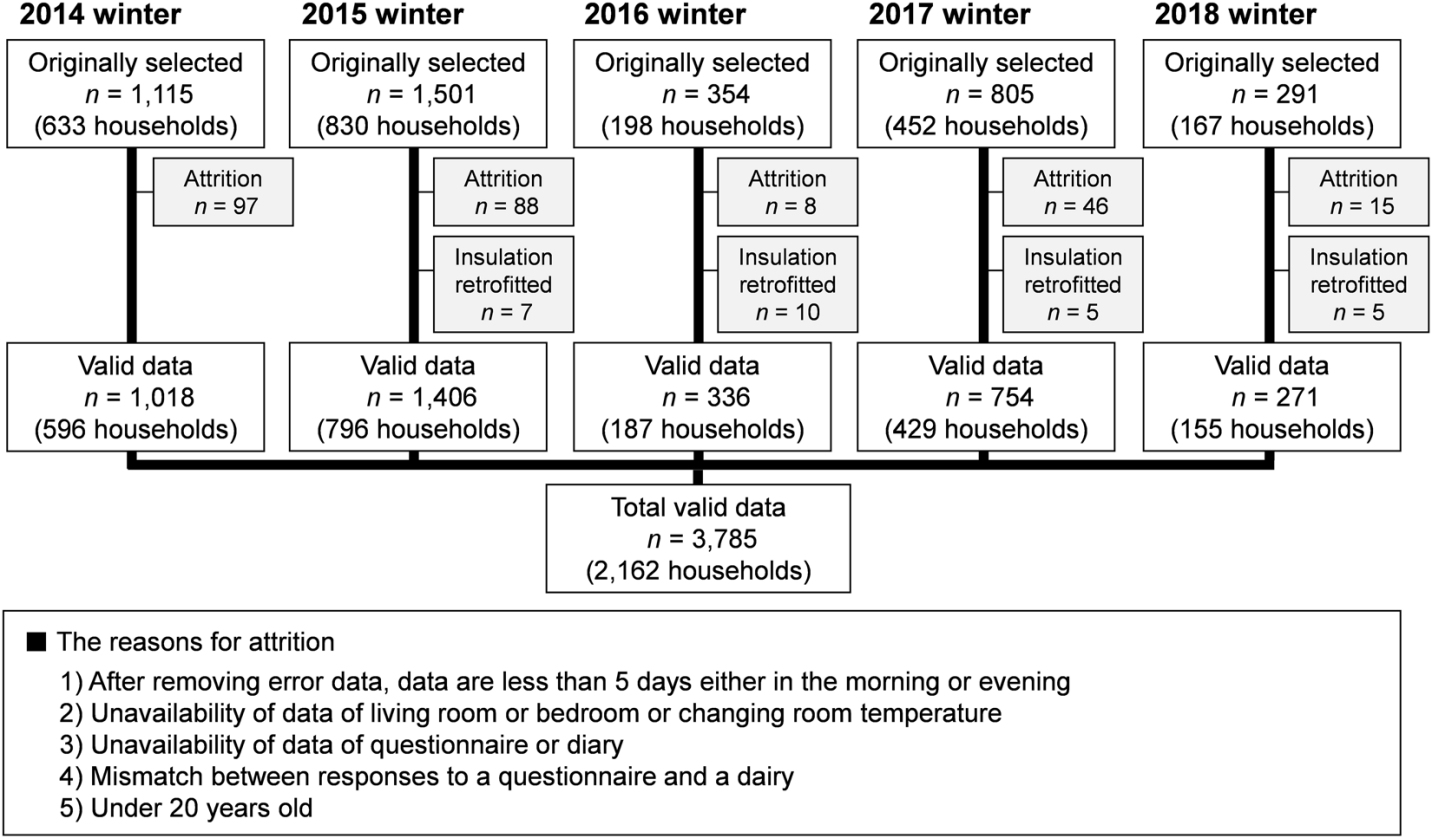
Flow chart of the selection of valid samples. “Insulation retrofitted” indicates participants who had already finished insulation retrofitting before the baseline survey. We excluded these samples because we analyzed data from the baseline (before insulation retrofitting) survey in this paper

Figure 1 shows the flow for selecting valid samples from the winter survey. Responses from 2,162 households and 3,785 participants were deemed valid. Table 1 shows the characteristics of the selected participants. The average age was 58 years (range: 20–99 years), 47% were men, and the average BMI of 22.8 kg/m^2^ was within the normal range indicated by the World Health Organization (WHO) (18.5–24.9 kg/m^2^). Approximately one-quarter of participants visited hospitals due to hypertension and used antihypertensive drugs.

**Table 1:** Baseline characteristics of participants in winter

Characteristic	
Individual attribute	
Age, *y* (SD)	58 (13)
Men, *n* (%)	1782 (47)
Body mass index, kg/m^2^ (SD)	22.8 (3.6)
High household income, *n* (%) (≥6 million JPY)	1319 (38)
Lifestyle	
Salt check sheet, points (SD)	13.1 (4.3)
Eating vegetable regularly, *n* (%)	2878 (77)
Current smoker, *n* (%)	508 (15)
Current drinker, *n* (%)	1975 (53)
Regular exercise, *n* (%)	1076 (29)
Antihypertensive drugs, *n* (%)	917 (25)
Health condition	
Stroke, *n* (%)	59 (2)
Angina/Myocardial infarction, n (%)	125 (3)
Hypertension, *n* (%)	878 (24)
Diabetes mellitus, *n* (%)	252 (7)
Hyperlipidemia, *n* (%)	652 (18)
Home blood pressure	
MEave of SBP, mmHg (SD)	126 (16)
MEave of DBP, mmHg (SD)	78 (10)
MEdif of SBP, mmHg (SD)	6.6 (10.1)
MEdif of DBP, mmHg (SD)	6.5 (6.7)
SD of SBP, mmHg (SD)	6.9 (2.7)
SD of DBP, mmHg (SD)	4.6 (1.9)
CV of SBP, % (SD)	5.4 (1.9)
CV of DBP, % (SD)	6.0 (2.2)
ARV of SBP, mmHg (SD)	7.5 (3.1)
ARV of DBP, mmHg (SD)	5.1 (2.2)
VIM of SBP, unit (SD)	6.8 (2.3)
VIM of DBP, unit (SD)	4.6 (1.7)
Temperature	
MEave of Temp_In_, °C (SD)	16.3 (3.1)
MEave of Temp_Out_, °C (SD)	4.3 (3.4)
MEdif of Temp_In_, °C (SD)	3.2 (2.4)
MEdif of Temp_Out_, °C (SD)	1.5 (1.1)
SD of Temp_In_, °C (SD)	1.6 (0.7)
SD of Temp_Out_, °C (SD)	2.5 (0.9)

Total number is 3785 participants (2162 households)

*MEave*  morning-evening average, *MEdif* morning-evening difference, *SD* standard deviation, *CV* coefficient of variation, *ARV* average real variability, *VIM* variability independent of the mean, *SBP* systolic blood pressure, *DBP* diastolic blood pressure *Temp*_*In*_, indoor ambient temperature, *Temp*_*Out*_ outdoor temperature

The average number of days of HBPM per participant was 13.6 (range: 5–14 days). The mean ME average systolic BP (SBP)/diastolic BP (DBP) was 126/78 mmHg. The mean ME difference in SBP/DBP, the diurnal variability index, was 6.6/6.5 mmHg. The mean SD, CV, ARV, and VIM of SBP/DBP, the day-by-day variability indices, were 6.9/4.6 mmHg, 5.4/6.0%, 7.5/5.1 mmHg, and 6.8/4.6 units, respectively.

**Fig. 2:**
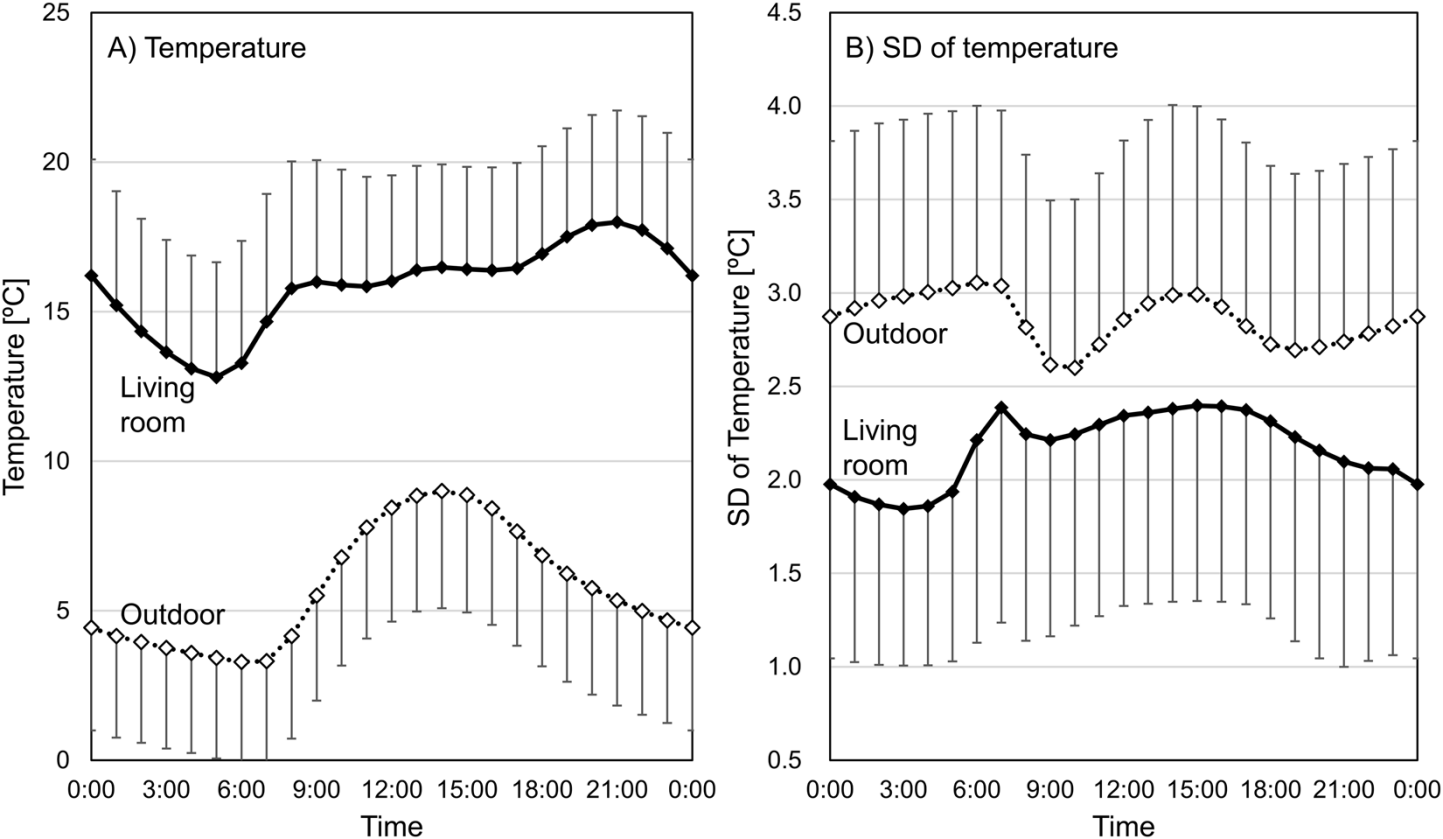
Fluctuation of room temperature and the SD of room temperature throughout a day. The solid line shows living room temperature, the dotted line shows outdoor temperature, and the error bar shows the standard deviation. The SD was calculated based on the temperature at the same hour for 2 weeks. Error bar indicates the variability among houses

The mean ME average indoor/outdoor temperature was 16.3 (range: 4.5–25.6 °C)/4.3 °C (range: −5.1–16.2 °C), which is below the recommended minimum home temperature of 18 °C determined by the WHO [22]. The mean ME difference in indoor/outdoor temperature, the diurnal instability index, was 3.2/1.5 °C, indicating that the decrease in indoor temperature overnight was greater than the decrease in outdoor temperature. The mean SD of indoor/outdoor temperature, a day-by-day instability index, was 1.6/2.5 °C. Figure 2 shows the fluctuation in living room and outdoor temperature and SD of these temperatures throughout the day. The average time at home was 17.3 h on average, indicating that participants spent a lot (72.0%) of time inside their houses. Living room temperature peaked (18.0 °C) at 9 PM before decreasing to a minimum (12.8 °C) as the outdoor temperature decreased. The SD of living room temperature increased from 5 AM to 7 AM.

### Diurnal and day-by-day HBP variability and indoor temperature instability

**Fig. 3:**
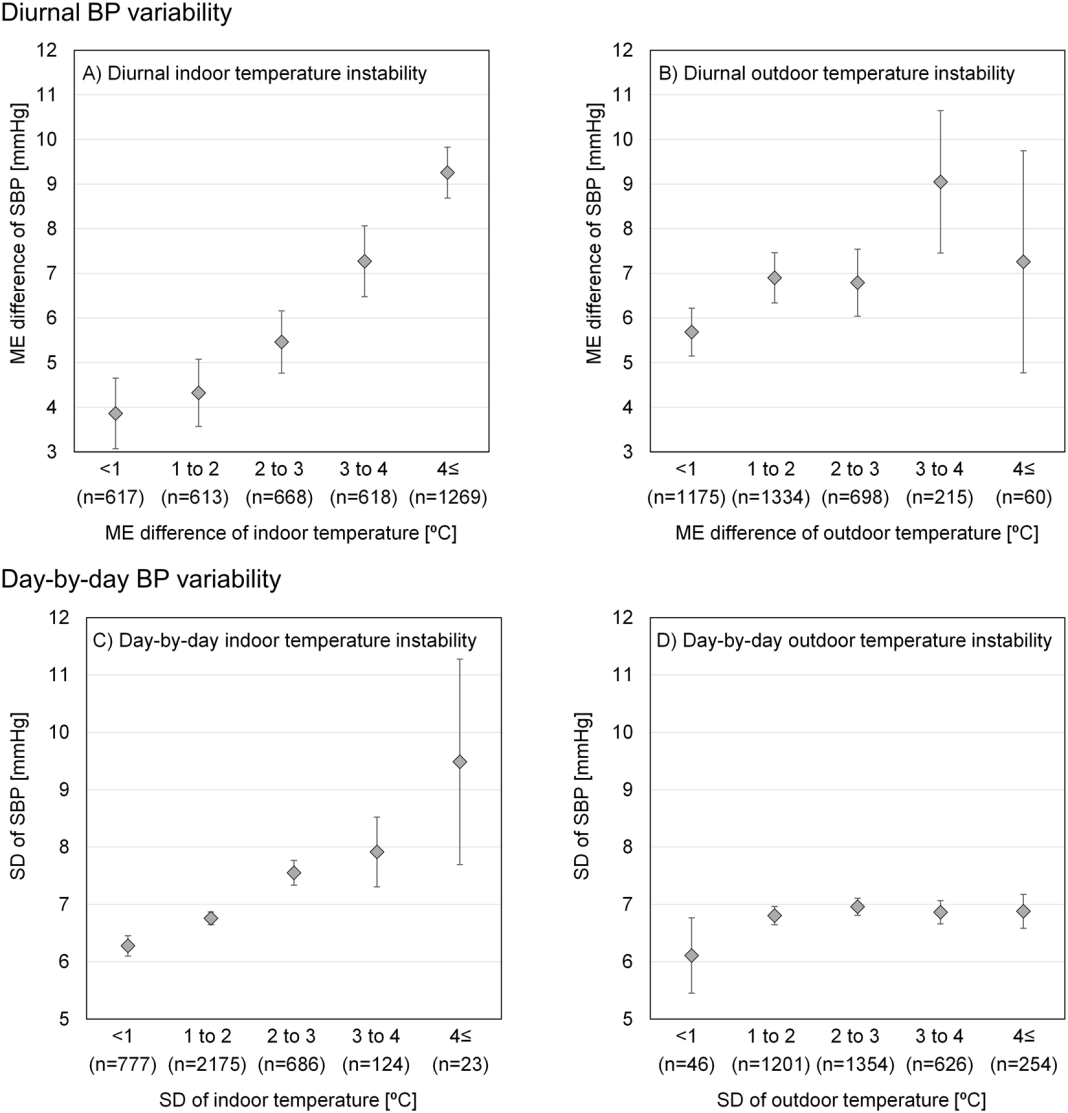
Relationship between BP variability and temperature instability. A: ME difference in SBP and ME difference in indoor temperature. B: ME difference in SBP and ME difference in outdoor temperature. C: SD of SBP and SD of indoor temperature. D: SD of SBP and SD of outdoor temperature. The plot shows the average of each group, and the error bar shows the 95% confidence interval

Figure 3A, B shows the mean ME difference in SBP in participants grouped based on the ME difference in indoor/outdoor temperature (a decrease in indoor/outdoor temperature overnight). There was a clear trend in which the ME difference in SBP increased with the ME difference in indoor temperature: the ME difference in SBP was 3.9 vs 9.3 mmHg (<1 °C vs ≥4 °C ME difference in indoor temperature). Figure 3C, D shows the mean SD of SBP in participants grouped according to the SD of indoor/outdoor temperature. While there was no correlation between the SD of SBP and outdoor temperature, there was a positive correlation between the SD of SBP and indoor temperature: the SD of SBP was 6.3 vs 9.5 mmHg (<1 °C vs ≥4 °C SD of indoor temperature).

Table 2 shows the results of multiple linear regression analysis of diurnal HBP variability. After adjusting for confounders, the ME difference in indoor temperature was significantly associated with the ME difference in BP (SBP: 0.85 mmHg/°C, DBP: 0.53 mmHg/°C). In contrast, the association with the ME difference in outdoor temperature observed in the univariate model was not significant in the multivariate model. Therefore, an ME difference in outdoor temperature showed a spurious correlation with an ME difference in BP in the univariate model. Table 3 shows the results of multiple linear regression analysis of day-by-day HBP variability indices. All day-by-day variability indices were positively associated with the SD of indoor temperature but not with the SD of outdoor temperature in the multivariate model. Thus, indoor temperature instability increases both diurnal and day-by-day HBP variability. We also analyzed data classified by climate area (Supplementary Fig. 3), which is defined by the Ministry of Land, Infrastructure, Transport and Tourism based on outdoor temperature and heating period. We performed the analyses in Areas 4–7, where 100 or more residents’ valid data had been obtained. A significant association between ME differences in HBP and ME differences in indoor temperature was observed in each area (Supplementary Table 2). The association between the SD of HBP and the SD of indoor temperature was also significant in each area (Supplementary Table 3).

**Table 2:** Multivariate analysis of the relationship between diurnal HBP variability and diurnal indoor temperature instability

Objective variable	Explanatory variable	Univariate model^a^			Multivariate model^b^			
		*β*	(95%CI)	*P* value	*β*	(95%CI)	Standard *β*	*P* value
MEdif of SBP	MEdif of Temp_In_	0.96	(0.83 to 1.09)	<0.001	0.85	(0.71 to 0.99)	0.22	<0.001
	MEdif of Temp_Out_	0.72	(0.41 to 1.03)	<0.001	−0.07	(−0.41 to 0.27)	−0.01	0.671
MEdif of DBP	MEdif of Temp_In_	0.52	(0.44 to 0.61)	<0.001	0.53	(0.43 to 0.62)	0.20	<0.001
	MEdif of Temp_Out_	0.28	(0.07 to 0.49)	0.008	−0.04	(−0.27 to 0.19)	−0.01	0.725

*CI*  confidence interval, *MEdif* morning-evening difference, *SBP* systolic blood pressure, *DBP* diastolic blood pressure, *Temp*_*In*_ indoor ambient temperature, *Temp*_*Out*_ outdoor temperature

^a^MEdif of Temp_In_ and MEdif of Temp_Out_ were independently put into the univariate model

^b^Adjusted for average Temp_In_, average Temp_Out_, average sleep quality, average sleep duration, age, sex, BMI, high household income, salt check sheet score, vegetable consumption, current smoker, current drinker, regular exercise, antihypertensive drug use

**Table 3:** Multivariate analysis of the relationship between day-by-day HBP variability and day-by-day indoor temperature instability

Objective variable	Explanatory variable	Univariate model^a^			Multivariate model^b^			
		*β*	(95%CI)	*P* value	*β*	(95%CI)	Standard *β*	*P* value
SD of SBP	SD of Temp_In_	0.75	(0.63 to 0.88)	<0.001	0.61	(0.47 to 0.75)	0.16	<0.001
	SD of Temp_Out_	0.07	(−0.03 to 0.17)	0.148	−0.03	(−0.15 to 0.08)	−0.01	0.564
CV of SBP	SD of Temp_In_	0.50	(0.42 to 0.59)	<0.001	0.44	(0.34 to 0.54)	0.17	<0.001
	SD of Temp_Out_	0.06	(−0.00 to 0.13)	0.067	−0.02	(−0.10 to 0.06)	−0.01	0.606
ARV of SBP	SD of Temp_In_	0.82	(0.68 to 0.96)	<0.001	0.64	(0.48 to 0.81)	0.15	<0.001
	SD of Temp_Out_	0.12	(0.00 to 0.23)	0.651	−0.01	(−0.15 to 0.12)	−0.00	0.879
VIM of SBP	SD of Temp_In_	0.58	(0.48 to 0.69)	<0.001	0.53	(0.40 to 0.66)	0.16	<0.001
	SD of Temp_Out_	0.08	(0.00 to 0.17)	0.048	−0.02	(−0.13 to 0.08)	−0.01	0.669
SD of DBP	SD of Temp_In_	0.51	(0.42 to 0.59)	<0.001	0.38	(0.27 to 0.48)	0.14	<0.001
	SD of Temp_Out_	0.06	(−0.01 to 0.12)	0.096	−0.02	(−0.10 to 0.06)	−0.01	0.634
CV of DBP	SD of Temp_In_	0.55	(0.45 to 0.65)	<0.001	0.41	(0.29 to 0.54)	0.13	<0.001
	SD of Temp_Out_	0.08	(0.01 to 0.16)	0.038	−0.02	(−0.12 to 0.08)	−0.01	0.703
ARV of DBP	SD of Temp_In_	0.54	(0.44 to 0.64)	<0.001	0.36	(0.24 to 0.47)	0.12	<0.001
	SD of Temp_Out_	0.07	(−0.01 to 0.15)	0.083	0.01	(−0.08 to 0.11)	0.01	0.789
VIM of DBP	SD of Temp_In_	0.42	(0.34 to 0.49)	<0.001	0.31	(0.22 to 0.41)	0.13	<0.001
	SD of Temp_Out_	0.07	(0.01 to 0.13)	0.034	−0.01	(−0.09 to 0.06)	−0.01	0.717

*CI*  confidence interval, *SD* standard deviation, *CV* coefficient of variation, *ARV* average real variability *VIM* variability independent of the mean, *SBP* systolic blood pressure, *DBP* diastolic blood pressure, *Temp*_*In*_ indoor ambient temperature, *Temp*_*Out*_ outdoor temperature

^a^SD of Temp_In_ and SD of Temp_Out_ were independently put into the univariate model

^b^Adjusted for average Temp_In_, average Temp_Out_, average and SD of sleep quality, average and SD of sleep duration, age, sex, BMI, high household income, salt check sheet score, vegetable consumption, current smoker, current drinker, regular exercise, antihypertensive drug use, the number of HBP measurement days

## Discussion

The key findings in the present study conducted on 2,162 households and 3,785 participants in Japan are as follows: (1) the mean ME difference in indoor/outdoor temperature, an index of diurnal instability, was 3.2/1.5 °C; (2) the mean SD of indoor/outdoor temperature, an index of day-by-day instability, was 1.6/2.5 °C; (3) compared to houses with a decrease in indoor temperature overnight (ME difference in indoor temperature) <1 °C, the ME difference in SBP was greater in houses with an ME difference in indoor temperature ≥4 °C (3.9 mmHg vs 9.3 mmHg); (4) compared to houses with an SD of indoor temperature <1 °C, the SD of SBP was greater in houses with an SD of indoor temperature ≥4 °C (6.3 mmHg vs 9.5 mmHg); and (5) after adjusting for confounders, diurnal and day-by-day BP variabilities were significantly associated with indoor temperature instability but not with outdoor temperature instability, indicating that indoor temperature instability increases both diurnal and day-by-day HBP variability. One of the possible reasons for the above findings is that thermophysiological reactions of blood vessels, such as vasoconstriction or vasodilatation, depend on the environmental temperature, leading to an increase or a decrease in BP.

### Clinical significance of diurnal and day-by-day HBP variability

The clinical significance of BP variability has not yet been established. Researchers have developed arguments about the pros [23] and cons [24] of BP variability, indicating that no consensus yet exists. However, there are many studies that support the significance of the BP variability indices used in the present study. In terms of diurnal HBP variability, previous research has shown that an ME difference in BP is associated with the left ventricular mass index [8]. In terms of day-by-day HBP variability, evidence from the Ohasama study [9, 25], the J-HOP study [10], the Finn-Home study [26], and a combination of several studies [27] indicates that high day-by-day HBP variability determined using measures such as the SD, CV, ARV, and VIM is associated with increased total, cardiovascular and stroke mortality, independent of average HBP level. A recent study [28] also clarified that the CV of HBP is associated with cognitive function. Furthermore, a systematic review [13] showed that HBP variability indices have an independent effect on the progression of cardiac organ damage and CVD events. A growing body of evidence indicates the significance of diurnal and day-by-day HBP variability. Thus, we believe BP variabilities assessed by HBPM are useful in evaluating the risk of CVD events.

### Determinants of diurnal HBP variability

Few studies have examined the determinants of this ME difference. Ishikawa et al. [11] showed that older age, *β*-blocker use, and regular alcohol consumption exaggerate the ME difference. Additionally, Ito et al. [12] revealed that drinking alcohol and bathing were associated with an increase in the ME difference. These studies also investigated ways to reduce the ME difference, focusing mainly on lifestyle factors. The present study showed that a decrease in indoor temperature overnight (ME difference of indoor temperature) was positively associated with the ME difference in BP. Although the circadian rhythm basically generates the ME difference in BP, the effect of temperature could be separated from that of circadian rhythm by comparing the ME difference in BP by indoor temperature instability groups. This result suggests that the combination of improving lifestyle habits and living environment is key to reducing diurnal HBP variability.

### Determinants of day-by-day HBP variability

Several factors have been shown to affect day-by-day variability, including individual attributes (e.g., older age, female sex, low BMI), lifestyle factors (e.g., excessive alcohol intake, smoking, sedentary lifestyle, *β*-blocker use) and health condition (e.g., a history of CVD and diabetes mellitus) [13]. However, there are currently no suggestions related to the living environment, such as the indoor thermal environment. In the present study, multivariate analyses indicated that day-by-day indoor temperature instability was positively correlated with day-by-day HBP variability. Thus, reducing day-by-day indoor temperature instability may be an effective way to reduce BP variability.

### Indoor temperature instability in Japan and other countries

The present study showed that indoor temperature decreased rapidly during the nighttime, and the SD of living room temperature increased in the morning. These results are attributed to the low level of insulation in most Japanese houses and the habit of Japanese to turn off heating devices before getting into bed and turn them on after getting out of bed. In Japan, 39% of approximately 50 million existing houses are not insulated, and only 5% of those are relatively well insulated to meet the 1999 standards (the highest thermal insulation standards) [16]. In addition, partial intermittent heating in the living room only is the norm in Japan. Although a previous report in Japan [29] described that heating equipment must be designed to allow residents to choose between continuous and partial intermittent heating, the latter is a common choice now because of the low insulation level of houses and energy inefficiency as a result. Thus, the combination of a high insulation level and continuous heating use is important to reduce both diurnal and day-by-day instability in indoor temperatures.

A few studies have investigated indoor temperature instability in other countries. In New Zealand, the mean ME difference in the living room temperature was 4.3 °C (mean evening temperature: 17.8 °C minus mean morning temperature: 13.5 °C) [30], which was larger than the 3.2 °C in the present study. In addition, in rural areas of China, the average daily variation amplitude of indoor temperature was 5.5 °C [31]. Although houses in European and American countries might have less indoor temperature instability because continuous heating is common there, indoor temperature instability might become a potential problem not only in Japan but also in other parts of the world, such as Asian and Oceanian countries.

### Recommendations for highly insulated houses and continuous heating to prevent CVD

Reducing both BP level and variability is considered important for the prevention of CVD events. Previous studies have shown the effect of heating and insulation retrofitting on BP levels. A randomized controlled trial [32] indicated that instruction in home heating reduced morning SBP by 4.4 mmHg. Furthermore, an intervention study [33] revealed that insulation retrofitting of houses significantly reduced morning home SBP by 3.1 mmHg. Based on these results, heating use and highly insulated houses are both important for reducing BP levels. However, from the viewpoint of BP variability, the use of heating devices can be both beneficial and harmful. While continuous heating contributes to keeping indoor temperatures stable, intermittent heating use results in diurnal indoor temperature instability, with the turning off of heating devices during sleep in particular leading to greater ME differences in indoor temperature. In addition, we found that heating use may increase day-by-day indoor temperature instability. In contrast, highly insulated houses are effective in reducing BP variability because they are less affected by outdoor temperature, resulting in indoor temperature stability. Therefore, we recommend prioritizing living in highly insulated houses over depending on heating use. We expect that these results will be useful for the prevention of CVD related to BP variability.

### Study limitations

The present study has several limitations that should be considered when interpreting our findings. First, because this survey was conducted on participants who intended to have their houses retrofitted with insulation, the study sample may be biased toward wealthy households. Furthermore, recommendations for HBPM differ between the Japanese guidelines and Western countries’ guidelines. For example, the timing of evening HBPM is “before getting into bed” in the Japanese guideline [18] and “before dinner” in the American and European guidelines [1, 34]. Therefore, the applicability of these findings should be considered with caution. Second, we were unable to obtain data on participants’ daily clothing use, which is a potential confounder for HBP variability. Further studies are needed to examine whether indoor temperature instability affects BP variability after adjusting for clothing use. Third, we could not obtain data on the exposure history of temperature, which might affect BP variability. Temperature inside the bed may also have a significant impact on BP variability, especially on diurnal variability. Therefore, a study design in which participants carry a temperature sensor in a wearable form is required to solve these challenges. Finally, we did not evaluate seasonal or yearly variability in BP due to the cross-sectional nature of the study. There is evidence [35, 36] that evaluates seasonal variability in BP and its relationship with temperature, and a review article has also been issued [37]. However, most previous research focused on the outdoor temperature, so the relationship between seasonal variation in BP and indoor temperature is still limited. Given findings that large seasonal variations in HBP are associated with CVD events [38] and target organ damage [39], the association between seasonal variation in BP and indoor temperature should be investigated in future studies. In terms of yearly variability, we hypothesize that a cold debt (living in a cold house for a long time) increases the yearly variability in BP. We plan to examine yearly BP variability in a long-term cohort study in cold and warm house groups (Supplementary Fig. 4).

### Perspectives

To our knowledge, this is the first epidemiological study to examine the association between diurnal and day-by-day BP variability and indoor temperature instability. The ME difference and day-by-day variability in BP can be reduced by keeping the indoor temperature stable. The present study provides new insight into the effects of improving one’s living environment, rather than simply improving lifestyle, on reducing BP variability. In addition, higher indoor temperatures at home also lower BP levels. Thus, indoor temperature may be a key factor for preventing CVD through its effects on both BP level and BP variability.

There are two main approaches to increasing indoor temperature as a means of reducing BP levels and decreasing indoor temperature instability as a means of reducing BP variability: living in a highly insulated house and using heating devices. While both approaches contribute to increasing the indoor temperature and decreasing indoor temperature instability, the use of heating devices is associated with added complications. This is because intermittent use of heating devices can cause increased indoor temperature instability. While the use of heating devices comes with strengths such as low initial cost and feasibility, living in a highly insulated house is preferable over depending on heating use for the prevention of CVD.

## Supplementary information

Supplementary information
