# Supplementary material for: Impact of indoor temperature instability on diurnal and day-by-day variability of home blood pressure in winter: a nationwide Smart Wellness Housing survey in Japan
Source: Hypertens Res. 2021 Jul 29;44(11):1406–16. doi: 10.1038/s41440-021-00699-x (PMC8568693; doi:10.1038/s41440-021-00699-x)
Supplement: Supplementary file 1 — Supplementary information [file 41440_2021_699_MOESM1_ESM.docx]

**Supplementary information**

Impact of Indoor Temperature Instability on Diurnal and Day-by-Day Variability of Home Blood Pressure in Winter: A Nationwide Smart Wellness Housing Survey in Japan

Wataru Umishio,^1,2^ Toshiharu Ikaga,^2^ Kazuomi Kario,^3^ Yoshihisa Fujino,^4^ Masaru Suzuki,^5^ Shintaro Ando,^6^ Tanji Hoshi,^7^ Takesumi Yoshimura,^8^ Hiroshi Yoshino,^9^ Shuzo Murakami^10^; on behalf of the SWH survey group

^1^Department of Architecture and Building Engineering, School of Environment and Society, Tokyo Institute of Technology, Ookayama, Meguro-ku, Tokyo, Japan

^2^Department of System Design Engineering, Faculty of Science and Technology, Keio University, Yokohama, Kanagawa, Japan

^3^Department of Cardiology, Jichi Medical University School of Medicine, Shimotsuke, Tochigi, Japan

^4^Department of Environmental Epidemiology, Institute of Industrial Ecological Sciences, University of Occupational and Environmental Health, Kitakyushu, Fukuoka, Japan

^5^Department of Emergency Medicine, Ichikawa General Hospital, Tokyo Dental College, Ichikawa, Chiba, Japan

^6^Department of Architecture, Faculty of Environmental Engineering, University of Kitakyushu, Kitakyushu, Fukuoka, Japan

^7^Tokyo Metropolitan University, Hachioji, Tokyo, Japan

^8^University of Occupational and Environmental Health, Kitakyushu, Fukuoka, Japan

^9^Tohoku University, Sendai, Miyagi, Japan

^10^Institute for Building Environment and Energy Conservation, Kojimachi, Chiyoda-ku, Tokyo, Japan

Supplementary Table 1. Average salt check sheet score in the present survey and salt intake in the National Health and Nutrition Survey by area

| **Area** | Hokkaido | Tohoku | Kanto I | Kanto II | Hokuriku | Tokai | Kinki I | Kinki II | Chugoku | Shikoku | Kita-kyushu | Minami-kyushu |
| --- | --- | --- | --- | --- | --- | --- | --- | --- | --- | --- | --- | --- |
| **Salt check sheet [pt]** | 13.2 | 14.4 | 13.5 | 13.0 | 13.7 | 13.1 | 12.2 | 12.7 | 13.3 | 12.7 | 12.1 | 12.9 |
| **Salt intake [g/day]** | 9.8 | 10.5 | 10.0 | 10.3 | 10.1 | 9.8 | 9.4 | 9.6 | 9.8 | 9.6 | 10.1 | 9.6 |

Summary of the results was as follows.

・Tohoku area had the highest value in the both indices.

・Correlation coefficient between two indices was significant (R=0.62, p=0.030).

・Regression line was "salt intake = 0.32 × salt check sheet score + 5.7."

Supplementary Table 2. Multivariate analysis of the relationship between diurnal HBP variability and

diurnal indoor temperature instability, classified by climate areas

| **Climate area** | **Objective variable** | **Explanatory**  **variable** |  | **Univariate model *** | | |  | **Multivariate model †** | | | |
| --- | --- | --- | --- | --- | --- | --- | --- | --- | --- | --- | --- |
|  |  |  |  | ***β*** | **(95%CI)** | ***P* value** |  | ***β*** | **(95%CI)** | **Standard *β*** | ***P* value** |
| All | MEdif of SBP | MEdif of Temp_In_ |  | 0.96 | (0.83 to 1.09) | <0.001 |  | 0.85 | (0.71 to 0.99) | 0.22 | <0.001 |
| (n=3785) |  | MEdif of Temp_Out_ |  | 0.72 | (0.41 to 1.03) | <0.001 |  | −0.07 | (−0.41 to 0.27) | −0.01 | 0.671 |
|  | MEdif of DBP | MEdif of Temp_In_ |  | 0.52 | (0.44 to 0.61) | <0.001 |  | 0.53 | (0.43 to 0.62) | 0.20 | <0.001 |
|  |  | MEdif of Temp_Out_ |  | 0.28 | (0.07 to 0.49) | 0.008 |  | −0.04 | (−0.27 to 0.19) | −0.01 | 0.725 |
| Area 4 | MEdif of SBP | MEdif of Temp_In_ |  | 1.05 | (0.65 to 1.45) | <0.001 |  | 1.06 | (0.63 to 1.48) | 0.26 | <0.001 |
| (n=424) |  | MEdif of Temp_Out_ |  | 1.19 | (−0.01 to 2.38) | 0.051 |  | 0.05 | (−1.29 to 1.40) | 0.00 | 0.939 |
|  | MEdif of DBP | MEdif of Temp_In_ |  | 0.58 | (0.31 to 0.85) | <0.001 |  | 0.66 | (0.37 to 0.95) | 0.24 | <0.001 |
|  |  | MEdif of Temp_Out_ |  | 0.48 | (−0.31 to 1.27) | 0.237 |  | −0.08 | (−1.00 to 0.85) | −0.01 | 0.867 |
| Area 5 | MEdif of SBP | MEdif of Temp_In_ |  | 0.77 | (0.52 to 1.03) | <0.001 |  | 0.60 | (0.32 to 0.88) | 0.15 | <0.001 |
| (n=995) |  | MEdif of Temp_Out_ |  | 1.57 | (1.00 to 2.14) | <0.001 |  | 0.67 | (0.06 to 1.28) | 0.08 | 0.031 |
|  | MEdif of DBP | MEdif of Temp_In_ |  | 0.41 | (0.23 to 0.58) | <0.001 |  | 0.35 | (0.16 to 0.54) | 0.13 | <0.001 |
|  |  | MEdif of Temp_Out_ |  | 0.60 | (0.21 to 0.99) | 0.003 |  | 0.29 | (−0.13 to 0.72) | 0.05 | 0.180 |

Supplementary Table 2. Multivariate analysis of the relationship between diurnal HBP variability and

diurnal indoor temperature instability, classified by climate areas (continued)

| **Climate area** | **Objective variable** | **Explanatory**  **variable** |  | **Univariate model *** | | |  | **Multivariate model †** | | | |
| --- | --- | --- | --- | --- | --- | --- | --- | --- | --- | --- | --- |
|  |  |  |  | ***β*** | **(95%CI)** | ***P* value** |  | ***β*** | **(95%CI)** | **Standard *β*** | ***P* value** |
| Area 6 | MEdif of SBP | MEdif of Temp_In_ |  | 1.00 | (0.82 to 1.17) | <0.001 |  | 0.87 | (0.67 to 1.06) | 0.23 | <0.001 |
| (n=1931) |  | MEdif of Temp_Out_ |  | 0.24 | (−0.21 to 0.68) | 0.303 |  | −0.56 | (−1.04 to −0.08) | −0.06 | 0.023 |
|  | MEdif of DBP | MEdif of Temp_In_ |  | 0.53 | (0.41 to 0.65) | <0.001 |  | 0.55 | (0.42 to 0.69) | 0.21 | <0.001 |
|  |  | MEdif of Temp_Out_ |  | 0.08 | (−0.22 to 0.37) | 0.617 |  | −0.25 | (−0.58 to 0.07) | −0.04 | 0.129 |
| Area 7 | MEdif of SBP | MEdif of Temp_In_ |  | 0.91 | (0.33 to 1.49) | 0.002 |  | 1.12 | (0.51 to 1.73) | 0.27 | <0.001 |
| (n=216) |  | MEdif of Temp_Out_ |  | 0.51 | (−0.65 to 1.67) | 0.387 |  | 0.15 | (−1.14 to 1.44) | 0.02 | 0.817 |
|  | MEdif of DBP | MEdif of Temp_In_ |  | 0.63 | (0.25 to 1.02) | 0.001 |  | 0.86 | (0.44 to 1.28) | 0.30 | <0.001 |
|  |  | MEdif of Temp_Out_ |  | 0.43 | (−0.34 to 1.21) | 0.273 |  | 0.67 | (−0.21 to 1.55) | 0.12 | 0.134 |

* MEdif of Temp_In_ and MEdif of Temp_Out_ were independently put into the univariate model.

† Adjusted for average Temp_In_, average Temp_Out_, average sleep quality, average sleep duration, age, sex, BMI, high household income, salt check sheet score, vegetable consumption, current smoker, current drinker, regular exercise, antihypertensive drug use.

CI, confidence interval; MEdif, morning-evening difference; SBP, systolic blood pressure; DBP, diastolic blood pressure; Temp_In_, indoor ambient temperature; Temp_Out_, outdoor temperature.

Supplementary Table 3. Multivariate analysis of the relationship between day-by-day HBP variability and

day-by-day indoor temperature instability**,** classified by climate areas

| **Climate area** | **Objective variable** | **Explanatory**  **variable** |  | **Univariate model *** | | |  | **Multivariate model †** | | | |
| --- | --- | --- | --- | --- | --- | --- | --- | --- | --- | --- | --- |
|  |  |  |  | ***β*** | **(95%CI)** | ***P* value** |  | ***β*** | **(95%CI)** | **Standard *β*** | ***P* value** |
| All | SD of SBP | SD of Temp_In_ |  | 0.75 | (0.63 to 0.88) | <0.001 |  | 0.61 | (0.47 to 0.75) | 0.16 | <0.001 |
| (n=3785) |  | SD of Temp_Out_ |  | 0.07 | (−0.03 to 0.17) | 0.148 |  | −0.03 | (−0.15 to 0.08) | −0.01 | 0.564 |
|  | SD of DBP | SD of Temp_In_ |  | 0.51 | (0.42 to 0.59) | <0.001 |  | 0.38 | (0.27 to 0.48) | 0.14 | <0.001 |
|  |  | SD of Temp_Out_ |  | 0.06 | (−0.01 to 0.12) | 0.096 |  | −0.02 | (−0.10 to 0.06) | −0.01 | 0.634 |
| Area 4 | SD of SBP | SD of Temp_In_ |  | 0.82 | (0.46 to 1.17) | <0.001 |  | 0.51 | (0.12 to 0.90) | 0.15 | 0.011 |
| (n=424) |  | SD of Temp_Out_ |  | 0.29 | (−0.05 to 0.63) | 0.090 |  | 0.39 | (0.01 to 0.77) | 0.13 | 0.046 |
|  | SD of DBP | SD of Temp_In_ |  | 0.76 | (0.51 to 1.01) | <0.001 |  | 0.53 | (0.21 to 0.84) | 0.19 | 0.001 |
|  |  | SD of Temp_Out_ |  | 0.23 | (−0.01 to 0.48) | 0.064 |  | 0.32 | (0.01 to 0.62) | 0.13 | 0.042 |
| Area 5 | SD of SBP | SD of Temp_In_ |  | 0.84 | (0.60 to 1.08) | <0.001 |  | 0.76 | (0.48 to 1.03) | 0.19 | <0.001 |
| (n=995) |  | SD of Temp_Out_ |  | 0.17 | (−0.02 to 0.36) | 0.073 |  | 0.02 | (−0.21 to 0.25) | 0.01 | 0.852 |
|  | SD of DBP | SD of Temp_In_ |  | 0.47 | (0.29 to 0.64) | <0.001 |  | 0.35 | (0.15 to 0.55) | 0.13 | <0.001 |
|  |  | SD of Temp_Out_ |  | 0.07 | (−0.06 to 0.20) | 0.280 |  | 0.05 | (−0.12 to 0.22) | 0.03 | 0.540 |

Supplementary Table 3. Multivariate analysis of the relationship between day-by-day HBP variability and

day-by-day indoor temperature instability**,** classified by climate areas (continued)

| **Climate area** | **Objective variable** | **Explanatory**  **variable** |  | **Univariate model *** | | |  | **Multivariate model †** | | | |
| --- | --- | --- | --- | --- | --- | --- | --- | --- | --- | --- | --- |
|  |  |  |  | ***β*** | **(95%CI)** | ***P* value** |  | ***β*** | **(95%CI)** | **Standard *β*** | ***P* value** |
| Area 6 | SD of SBP | SD of Temp_In_ |  | 0.71 | (0.54 to 0.89) | <0.001 |  | 0.52 | (0.31 to 0.73) | 0.13 | <0.001 |
| (n=1931) |  | SD of Temp_Out_ |  | 0.03 | (−0.11 to 0.18) | 0.637 |  | −0.11 | (−0.28 to 0.05) | −0.04 | 0.178 |
|  | SD of DBP | SD of Temp_In_ |  | 0.47 | (0.35 to 0.59) | <0.001 |  | 0.37 | (0.22 to 0.52) | 0.14 | <0.001 |
|  |  | SD of Temp_Out_ |  | 0.07 | (−0.03 to 0.16) | 0.162 |  | −0.06 | (−0.18 to 0.06) | −0.03 | 0.325 |
| Area 7 | SD of SBP | SD of Temp_In_ |  | 0.70 | (0.14 to 1.26) | 0.015 |  | 0.96 | (0.17 to 1.76) | 0.22 | 0.017 |
| (n=216) |  | SD of Temp_Out_ |  | −0.07 | (−0.44 to 0.31) | 0.732 |  | −0.01 | (−0.53 to 0.52) | −0.00 | 0.984 |
|  | SD of DBP | SD of Temp_In_ |  | 0.67 | (0.29 to 1.04) | <0.001 |  | 1.00 | (0.48 to 1.52) | 0.35 | <0.001 |
|  |  | SD of Temp_Out_ |  | −0.07 | (−0.32 to 0.18) | 0.580 |  | −0.31 | (−0.66 to 0.03) | −0.16 | 0.073 |

* SD of Temp_In_ and SD of Temp_Out_ were independently put into the univariate model.

† Adjusted for average Temp_In_, average Temp_Out_, average and SD of sleep quality, average and SD of sleep duration, age, sex, BMI, high household income, salt check sheet score, vegetable consumption, current smoker, current drinker, regular exercise, antihypertensive drug use, the number of HBP measurement days.

CI, confidence interval; SD, standard deviation; SBP, systolic blood pressure; DBP, diastolic blood pressure; Temp_In_, indoor ambient temperature; Temp_Out_, outdoor temperature.

Supplementary Table 4. Members of Smart Wellness Housing Survey Group

A: Members of the Research Committee for the Promotion of Smart Wellness Housing

| **Chairperson** | |
| --- | --- |
| Shuzo MURAKAMI * | Institute for Building Environment and Energy Conservation |
| **Vice-chairperson** | |
| Takesumi YOSHIMURA * | University of Occupational and Environmental Health |
| Hiroshi YOSHINO * | Tohoku University |
| Kazuomi KARIO * | Jichi Medical University |
| **Organizer** | |
| Toshiharu IKAGA * | Keio University |
| **Committee member in medicine** | |
| Suminori AKIBA | Kagoshima University |
| Mikio ARITA | Sumiya Rehabilitation Hospital |
| Michiya IGASE | Ehime University |
| Masayoshi ICHIBA | Saga University |
| Nami IMAI | Mie University |
| Masaki UEMURA | At Home, LLC |
| Hiroyuki UEHARA | National Assembly Promoting Healthy and Energy Conserving Housing |
| Haruo UGUISU | Tokushima Bunri University |
| Kensuke ESATO | Yamaguchi University |
| Akira EBOSHIDA | Hiroshima University |
| Yuko OGUMA | Keio University |
| Toshiyuki OJIMA | Hamamatsu University School of Medicine |
| Shimato ONO | Marugame Ono Clinic |
| Yoshio OMATA | Hoju, Co., Ltd. |
| Takahiko KATOH | Kumamoto University |
| Masahiko KATO | Tottori University |
| Shinya KUNO | University of Tsukuba |
| Kiyokage KUBO | Kubo Clinic |
| Yoshiki KURODA | University of Miyazaki |
| Yasuaki SAIJO | Asahikawa Medical University |
| Kazuhiro SATO | University of Fukui |
| Eiji SHIBATA | Yokkaichi Nursing and Medical Care University |
| Kuninori SHIWAKU | Shimane University |
| Narufumi SUGANUMA | Kochi University |

Supplementary Table 4. Members of Smart Wellness Housing Survey Group

A: Members of the Research Committee for the Promotion of Smart Wellness Housing (continued)

| **Committee member in medicine (continued)** | |
| --- | --- |
| Tomotaka SOBUE | Osaka University |
| Toshiro TAKEZAKI | Kagoshima University |
| Masatoshi TANAKA | Fukushima Medical University |
| Tsuyoshi TANABE | Yamaguchi University |
| Susumu TSUKAMOTO | Saitama Jikei Hospital |
| Hiroyuki DOI | Okayama University |
| Kunio DOBASHI | Jobu Hospital for Respiratory Diseases |
| Chisato NAGATA | Gifu University |
| Hiroyuki NAKAMURA | Kanazawa University |
| Kunio NAKAYAMA | Former Osaka University |
| Norihiro NOGATA | Saiseikai Karatsu Hospital |
| Takashi HANATO | Eigenji Clinic |
| Yoshihisa FUJINO * | University of Occupational and Environmental Health |
| Tanji HOSHI * | Tokyo Metropolitan University |
| Satoshi HOSHIDE | Jichi Medical University |
| Takahiro MAEDA | Nagasaki University |
| Muneo MINOSHIMA | Minoshima Clinic |
| Takashi MURAWAKA | Yumemokuba, SNPC |
| Hidekazu YAMADA | Kindai University Nara Hospital |
| Misako YOSHINAGA | Kusunoki Hospital |
| **Committee member in architecture** | |
| Akihiko IWASA | Hosei University |
| Atsushi IWAMAE * | Kindai University |
| Akihito OZAKI | Kyushu University |
| Satoru KUNO | Nagoya University |
| Minoru KUMANO | Miyazaki University |
| Shoichi KOJIMA | Saga University |
| Yasuyuki SHIRAISHI | University of Kitakyushu |
| Hirotaka SUZUKI | Hokkaido Research Organization |
| Tsuyoshi SEIKE * | Tokyo University |
| Naoki TAKAGI | Shinshu University |

Supplementary Table 4. Members of Smart Wellness Housing Survey Group

A: Members of the Research Committee for the Promotion of Smart Wellness Housing (continued)

| **Committee member in architecture (continued)** | |
| --- | --- |
| Masaki TAJIMA | Kochi University of Technology |
| Yoshito TANAKA | Nagasaki Institute of Applied Science |
| Takayuki TAMAI | National Institute of Technology, Yonago College |
| Mitsutaka TSUJI | Gifu Academy of Forest Science and Culture |
| Reiji TOMIKU | Oita University |
| Hisaya NAGAI | Mie University |
| Daisaku NISHINA | Hiroshima University |
| Hideyo NIMIYA | Kagoshima University |
| Kenichi HASEGAWA | Akita Prefectural University |
| Hirofumi HAYAMA * | Hokkaido University |
| Akira FUKUSHIMA | Former Hokkaido University of Science |
| Yuji HORI | University of Toyama |
| Takeo MATSUOKA | Asia University |
| Teruaki MITAMURA | Maebashi Institute of Technology |
| Shinji YOSHIDA | Nara Women's University |

*: members of the Research Planning Committee for the Promotion of Smart Wellness Housing

B: Members of the Subcommittee for Analysis of the Smart Wellness Housing Survey

| **Chairperson** | |
| --- | --- |
| Toshiharu IKAGA * | Keio University |
| **Vice-chairperson** | |
| Yoshihisa FUJINO * | University of Occupational and Environmental Health |
| **Organizer** | |
| Shintaro ANDO * | University of Kitakyushu |
| Tatsuhiko KUBO | Hiroshima University |
| **Committee member** | |
| Wataru UMISHIO | Tokyo Institute of Technology |
| Yuko OGUMA | Keio University |
| Naoki KAGI | Tokyo Institute of Technology |
| Hiroshi KANEGAE | Genki Plaza Medical Center for Health Care |
| Shun KAWAKUBO | Hosei University |
| Yoshinobu SAITO | Kanagawa University of Human Services |
| Keigo SAEKI | Nara Medical University |
| Masaru SUZUKI | Tokyo Dental College Ichikawa General Hospital |
| Tsuyoshi SEIKE * | Tokyo University |
| Takayuki TAJIMA | Tokyo Metropolitan University |
| **Experts committee member** | |
| Maki ITO | Japan Federation of Housing Organizations |
| Hiroshi KOJIMA | Keio University |
| Natsue DOIHARA | Keio University |
| **Adviser** | |
| Takesumi YOSHIMURA * | University of Occupational and Environmental Health |
| Kazuomi KARIO * | Jichi Medical University |
| Tanji HOSHI * | Tokyo Metropolitan University |

*: members of the Research Planning Committee for the Promotion of Smart Wellness Housing

Supplementary Figure 1. Definition of morning-evening difference (MEdif) and

average (MEave) of BP

Supplementary Figure 2. Definition of day-by-day variability indices of BP

SD, standard deviation; CV, coefficient of variation; ARV, average real variability, VIM, variability independent of the mean

Morning-evening average of BP was used as BP value of each day (BP_i_)

Supplementary Figure 3. Eight climate areas in Japan as of 2014

Supplementary Figure 4. Overview of the before and after insulation retrofitting survey in Japan

(1) Study before and after insulation (validation α)

This is a before and after study to investigate short-term changes in indoor environment and health condition before and after insulation retrofitting.

(2) Study of long-term cohort (validation β)

This is a cohort study to verify the difference in long-term health effects due to differences in the adiabatic level by conducting a follow-up survey on health data of households after completion of insulation retrofitting. For households that do not carry out insulation retrofitting, these are also randomly selected and investigated as a control group.
